# The Vascularized Fibular Graft in the Pediatric Upper Extremity: A Durable, Biological Solution to Large Oncologic Defects

**DOI:** 10.1155/2013/321201

**Published:** 2013-10-07

**Authors:** Nicki Zelenski, Brian E. Brigman, L. Scott Levin, Detlev Erdmann, William C. Eward

**Affiliations:** ^1^Department of Orthopaedic Surgery, Duke University Medical Center, 200 Trent Drive, P.O. Box 2923, Durham, NC 27710, USA; ^2^Department of Orthopaedic Surgery, Hospital of the University of Pennsylvania, Philadelphia, PA 19104, USA; ^3^Department of Plastic and Reconstructive Surgery, Department of Surgery, Duke University Medical Center, Durham, NC 27710, USA

## Abstract

Skeletal reconstruction after large tumor resection is challenging. The free vascularized fibular graft (FVFG) offers the potential for rapid autograft incorporation as well as growing physeal transfer in pediatric patients. We retrospectively reviewed eleven pediatric patients treated with FVFG reconstructions of the upper extremity after tumor resection. Eight male and three female patients were identified, including four who underwent epiphyseal transfer. All eleven patients retained a functional salvaged limb. Nonunion and graft fracture were the most common complications relating to graft site (27%). Peroneal nerve palsy occurred in 4/11 patients, all of whom received epiphyseal transfer. Patients receiving epiphyseal transplant had a mean annual growth of 1.7 cm/year. Mean graft hypertrophy index increased by more than 10% in all cases. Although a high complication rate may be anticipated, the free vascularized fibula may be used to reconstruct large skeletal defects in the pediatric upper extremity after oncologic resection. Transferring the vascularized physis is a viable option when longitudinal growth is desired.

## 1. Introduction

Many patients who undergo resection of primary malignant bone tumors of the extremity are skeletally immature [1]. Current adjuvant chemotherapy and radiation regimens have increased the survival of many of these patients such that any reconstruction performed must be durable over time [2]. Limb salvage surgery has replaced amputation as the standard of care in most of these patients [3]. The high functional demands, need for longitudinal growth, and expected longevity of a salvaged pediatric limb pose unique problems to the reconstructive orthopaedic surgeon. 

There are a number of techniques which have the potential for success including endoprosthesis, allografts, and autografts—both avascular or vascular [4]. The decision of which technique to utilize depends on tumor-related factors such as size and location, as well as patient and surgeon related factors [4]. The goals of long term fixation and the need for high functionality render conventional endoprostheses suboptimal in the pediatric patient. Additionally, many endoprostheses are not available in sizes to fit small children. Osteoarticular allografts and endoprostheses also may be more susceptible to complications such as infection, aseptic loosening, and implant failure [5–9]. Avascular autografts heal by creeping substitution—a simultaneous process of osteoclastic and osteogenic activity—which weakens grafts and makes them susceptible to nonunion, delayed union, and fracture; as such these are typically restricted to use in defects smaller than 6 cm [10–12]. Vascularised autografts retain their biologic and mechanical properties and heal by primary union [13]. As a result, they can be used in defects up to 26 cm in length [14] as well as in the poorly vascularized tissue beds seen in pediatric patients undergoing radiation or chemotherapy [15]. 

The free vascularized fibular graft (FVFG) has become the most commonly utilized vascular autograft for segmental bone defects after trauma, nonunion, pseudarthrosis, osteonecrosis, and tumor resection [1, 16, 17]. FVFG transfer was first described in 1975 by Taylor et al. in two cases of lower extremity trauma [18]. Weiland et al. later performed this procedure in long bones for segmental skeletal defects after tumor resection [19]. The FVFG has greater structural application as well as lower donor site morbidity than vascularized rib or iliac crest grafts [20]. Additionally, it offers the opportunity for growing physeal transfer in pediatric patients. The viable physis and epiphysis in proximal FVFG allow for longitudinal growth as well as remodeling potential of the articular surface [15]. Innocenti first described this procedure in 1998 in children <10 years of age [21]. The average longitudinal growth rate of grafts was approximately 1 cm annually (range 0.75 to 1.33 cm). 

When harvesting the fibula for physeal transfer, the epiphysis, physis, and variable amounts of diaphysis are harvested, often along with the anterior tibial artery as the vascular pedicle, although it is somewhat controversial which artery to use [15]. The dual blood supply of nutrient endosteal vessels and periosteal vessels renders it amendable to transverse and longitudinal splitting [20]. The epiphysis and proximal diaphysis are supplied by branches of the anterior tibial artery, so there is no need to perform a double pedicle anastomosis to a proximal fibular graft [15]. The peroneal artery supplies the middle third of the fibula. 

Reconstructions using FVFG result in a construct with the potential for rapid union which is more resistant to infection than allografts [22]. Additionally, physeal transfers introduce the potential for longitudinal growth and joint remodeling in young patients. Although there is much literature describing FVFG for reconstruction after oncologic resection, there is little data on reconstruction of the upper extremity in the pediatric population and even less on physeal transfers in these patients. Based on our clinical experience with 11 free vascularized fibular grafts for reconstruction of upper extremity defects after oncological resection, 4 of which are physeal transfers, we investigated limb survival, graft union, graft fracture, longitudinal growth, and hypertrophy index in this unique patient population. 

## 2. Patients and Methods

We retrospectively reviewed our records for FVFG reconstructions of skeletal defects of the upper extremity after tumor resection in the pediatric population. Patients were identified from oncology and reconstructive surgery databases, and medical records were reviewed. We recorded patient demographics, primary diagnosis, location of malignancy, presence of metastatic disease, survivorship, adjuvant therapy, presence of local recurrence, complications, operative procedure and hardware used, time to union, additional operations required, longitudinal growth, and graft hypertrophy. Eight male and three female patients were identified, including four who underwent epiphyseal transfer. Mean age was 10.1 (range, 6–17 years). All eleven cases were primary bone tumors which included osteosarcoma (*n* = 6), osteosarcoma telangiectasia (*n* = 1), myxoid chondrosarcoma (*n* = 1), giant cell tumor (*n* = 2), and Ewing's sarcoma (*n* = 1). Sites of resection and reconstruction included distal radius (*n* = 2), ulna (*n* = 1), and humerus (*n* = 8). Nine patients received preoperative chemotherapy and none received radiation. Minimum followup was one year (mean: 3.3, range: 1–13 years). No patients were recalled specifically for this chart review.

Wide resection of the primary tumor was attempted in all cases. 9 FVFG reconstructions were performed at the time of tumor resection and two were performed at a later date. There were four osteocutaneous and seven osseous fibular grafts. Resections were performed by one of three orthopaedic surgeons (Brian E. Brigman, L. Scott Levin or William C. Eward). Inset of FVFG was performed by one of two orthopaedic or plastic surgeons (L. Scott Levin or Detlev Erdmann). Resection and inset are as previously described at this institution [10, 23] and included standard technique of fibula harvest [23–25] and end-to-end arterial and venous anastomosis [20]. In each case involving epiphyseal transfer, the anterior tibial artery was utilized as the donor artery. The peroneal artery was not utilized for any of our epiphyseal transfers (*n* = 4). The peroneal artery was utilized as the donor artery for each of the diaphyseal fibular transfers (*n* = 7). When an osteocutanous flap was not used, Cook implantable Doppler probes (cook Vascular Inc., Vandergrift, PA) were placed into recipient vessels at the time of surgery and removed before the patient was discharged. The mean length of the skeletal defect after resection was 14.8 cm. The mean length of the FVFG was also 14.8 cm. Osteosynthesis involved plate and screws (*n* = 4), external fixation (*n* = 1), screws alone (*n* = 3), plate, screws and joint fusion (*n* = 2), and Kirschner wire (*n* = 1) (Table 1). For the proximal humeral reconstructions, the soft tissue remnants of the proximal fibula (e.g., biceps femoris, lateral collateral ligament) were hand-sewn directly to the soft tissue remnants of the shoulder joint capsule and rotator cuff. Redundant soft tissues surrounding the shoulder joint were then imbricated to surround the fibular head. For the patient with reconstruction about the wrist, a radiocarpal arthrodesis was performed with plate and screw fixation distally into the scaphoid and capitate bones. 

Clinical followup occurred 2 weeks postoperatively for suture/staple removal and wound inspection, 6 weeks postoperatively, and then every 4 to 8 weeks until osseous union was observed radiographically. With the exception of the 2 week followup (where radiographs are not obtained), radiographs were obtained at every followup visit. For this study, all postoperative radiographs were evaluated by a single orthopedic surgeon (William C. Eward) for evidence of union, hypertrophy, longitudinal growth, and fracture. Osseous union was defined as described by Gebert et al. and included attenuation or absence of osteotomy line, presence of external bridging callus, or bony trabeculae spanning the osteosynthesis site [16]. We assessed hypertrophy of fibula graft using the DeBoer and Wood graft hypertrophy index [26]. Hypertrophy index for physeal transfers was calculated at site of bony union, either proximal or distal to physis. Otherwise, hypertrophy index was calculated at the distal osteosynthesis site.

 Consider

(1) $$\begin{array}{c} x\%\;\text{Hypertrophy}=\frac{{\text{index}}^{2}-{\text{index}}^{1}}{{\text{index}}^{1}}\times 100, \end{array}$$

where

(2) index1=diameter  of  the  graft  at  operationdiameter  of  recipient  bone  soon  after  operation,index2=diameter  of  the  graft  at  followup  diameter  of  recipient  bone  at  followup.

## 3. Results

All eleven patients retained a functional salvaged limb during the followup period. Of the four osteocutaneous grafts, all were deemed living at their most recent encounter by validity of skin pedicle. Eight patients had a total of 11 complications (73%), six of which required reoperation (3 for nonunions, 1 for nonunion of fracture of graft, 1 I&D, and 1 for hypertrophic scar) (Table 2). Nonunion and graft fracture were the most common complications relating to graft site (27%). Union was ultimately attained in all eleven grafts. Mean time to union after primary operation in patients not requiring reoperation was 7.7 months. Three patients (27%) developed nonunion, defined in this study as no clear evidence of bony union at six months postoperative and without evidence of progressive incorporation occurring. A single reoperation achieved successful union in all of these patients and consisted of revision with compression plate fixation and bone grafting. Mean time to union in these patients requiring reoperation for nonunion was 7 months after secondary procedure. Ten of the patients in this series were free of disease at the time of their last follow-up visit (mean = 57 months) while one patient with osteosarcoma had developed pulmonary metastases by her last follow-up visit.

**Figure 1:**
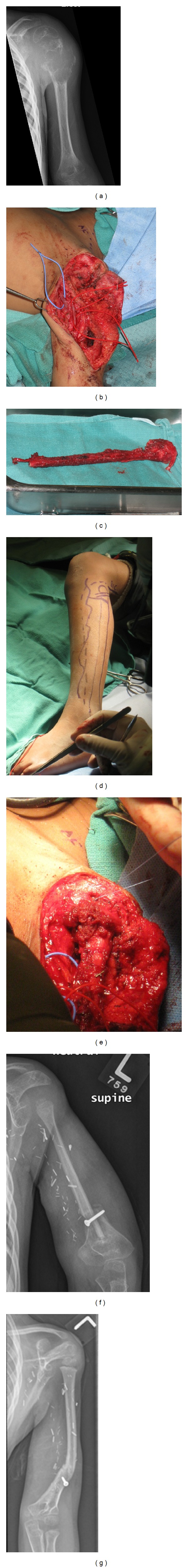
Operative pictures and nonoperative treatment of graft fracture. (a) Preoperative radiograph of osteosarcoma involving proximal humerus. (b) Intraoperative picture of resected tumor bed. (c) Resected tumor and proximal humerus. (d) Intraoperative planning of fibular resection. (e) Intraoperative reconstruction of proximal humerus with proximal fibula including growth plate. (f) AP radiograph immediately after operation. (g) Healed fracture through fibular graft with nonoperative treatment.

Fracture was also a common complication, with three patients fracturing through their graft (27%). One of these patients developed nonunion of the fracture site and required two reoperations to achieve union. The other two were treated successfully with nonoperative intervention (Figures 1(f) and 1(g)). Fracture occurred in the humerus in all three cases. Although one patient had wound breakdown, there was no evidence of infection upon I&D, and no cases of infection were confirmed in any of these patients. 

None of the diaphyseal FVFG transfers had defects at the graft site; however, all 4/4 of the epiphyseal transfers had peroneal nerve defects making it the most common complication overall (36%). This was evidenced by weakness/loss of ankle dorsiflexion and eversion as well as sensory loss on the dorsum of the foot. 1/4 patients resolved within 3 months and was left with no defects, 2/4 were left with residual foot drop but did not require AFO or assistive device for ambulation, and 1/4 required AFO at the date of last followup.

Evidence of longitudinal growth and hypertrophy was evaluated for these patients over the followup period. Patients receiving a FVFG without transfer of the proximal fibular epiphysis had an average annual growth of −3.7 mm. Those with epiphyseal transplant had an average annual growth of 17 mm with a mean growth of 26.4 mm total (9.6–62.4 mm). Mean graft hypertrophy index increased by more than 10% in all cases and was similar between epiphyseal and nonepiphyseal FVFG transfers (53.2% and 55.7%, resp.) and ranged from 11.2%–142%. In reconstruction of the humerus, the mean hypertrophy index was 61.8% (16.2–142%). In reconstruction of the forearm, the mean hypertrophy index was markedly lower with a mean of 32.2% (21.3–43%) (Table 3). Three patients predated accurate radiographic measuring tools such that radiological technology at the time of their follow-up visits did not permit calculation of a graft hypertrophy index or longitudinal growth. However, bony union was confirmed as well as hypertrophy at both the proximal and distal osteosynthesis sites.

## 4. Discussion

Skeletal reconstruction of large oncologic defects remains challenging in the pediatric population. The premium on durability of the reconstruction makes biological reconstruction more desirable than endoprosthetic reconstruction. Allografts can only be used in small defects and their failure often leads to limb loss [27]. Free vascularized fibular transfer offers the potential for rapid autograft incorporation in patients undergoing adjuvant chemotherapy or radiation. Furthermore, they can be used in large skeletal defects as they retain their biological and mechanical properties while they heal by primary union [13]. There has been a paucity of literature describing the outcomes of FVFG in the pediatric upper extremity. In the present study, we reconstructed 11 defects between 7 and 20 cm in length in pediatric patients after resection of malignant tumors. Limb salvage was achieved in all cases.

Like all reconstruction of large skeletal defects, FVFG has a high complication rate. We found an overall complication rate of 63% which is similar to other studies (37–80%) [10, 16, 22, 28, 29]. Fifty-four percent of patients required at least one additional operation. Nonunion and graft fracture were the most common complications (27% each). This is similar in comparison to two studies of FVFG in the upper extremity in which fracture was the most common complication [16, 28]. Rates of nonunion of FVFG have been reported as less common in several studies [16, 22, 28, 29]. Nonunion has been dismissed by some authors as unlikely when graft viability endures [15]. However, the method of assessing graft viability (visualization of skin paddle, Doppler, and nuclear scintigraphy) is often unclear in these reports. In the present study, skin pedicles were alive in all 4 osteocutaneous grafts, two of which developed nonunion. The high rate of nonunion in our study regardless of graft viability is high relative to other small published series but similar to a previous study at this institution [10]. All of our current patients with nonunion initially underwent fixation without compression with plate and screws in noncompression or simply K-wire. When revised with dynamic compression plate (2/3) or locking plate (1/3) with bone graft, all three went on to heal with mean time to union after secondary operation being 7 months. This suggests that the use of compression fixation during the index procedure would be advantageous for union in these patients.

Graft fracture occurred at the same rate in our patients as nonunion (27%). This is similar to a similar study of FVFG in the upper extremity in adults where fracture rate was 24% [16]. In our series, one patient fractured after a traumatic fall and went onto nonunion requiring two surgeries. His fracture ultimately healed 26 months after the second operation. The other two nontraumatic fractures, which occurred in the early postoperative period, were managed successfully nonoperatively. All fractures occurred in humeri with no fractures in the radius or ulna. This is consistent with the observations of Gebert et al. who reported that 80% of fractures in a series reporting FVFG in the upper extremity involved the humerus [16]. We find that fractures occurring in the late postoperative period are generally more challenging to treat than fractures in the early postoperative period. The large difference in diameter between fibula and humerus likely plays a role in the development of this complication in this location. In the femur, where this size mismatch is even greater, we have described the use of a larger allograft attached to the end of the fibula to render osteosynthesis more facile [30]. Perhaps this technique should be entertained when the humerus-to-fibula size mismatch is significant. Graft fracture is an important complication as it alters rehabilitation and is at least theoretically preventable. It is possible our fracture rate is higher than similar studies in adults due to high functional demands and low compliance in children. This is supported by Gebert et al. who reported an increase in graft fracture in the younger population of his patients [16]. All graft fractures ultimately healed and resulted in successful limb salvage.

An exciting advantage of vascularized epiphyseal transfer is the potential for longitudinal growth until physeal closure at skeletal maturity. We had excellent growth in all of our physeal transfers. There are several published case reports of vascularized epiphyseal transplant; however, few series of more than two patients exist [21, 31–34]. In 2007, Innocenti et al. reported the only large series—27 cases—of vascularized epiphyseal transfer to the upper extremity [35]. He reports fractures almost exclusively in the humerus (5/17 humeral cases) as well as annual growth rates similar to those obtained at our institution (0.7–1.35 cm/year, 1.72 cm/year, resp.). Two of four of our vascularized epiphyseal transplants fractured through their graft, which is higher than the diaphyseal transplant fracture rate (1/7); however, both were managed nonoperatively and progressed to union with no impact on growth. Peroneal nerve palsy occurred in 4/11 FVFG patients, all of whom received epiphyseal transfer. Due to the proximity of the peroneal nerve, peroneal palsy is common and is reported to occur in half of patients who undergo proximal fibula harvesting [15]. However, as in our cases, most are reported to resolve or improve with time [36]. Because limb length discrepancy is well tolerated in the upper extremity, we did not obtain radiographs of the contralateral limb to evaluate symmetry. Such data would be useful in future studies.

The mean hypertrophy index of the forearm was markedly lower than that of the humerus, and as addressed above, the rate of complication was much higher in humerus. This is supported by results in Gebert et al., who speculates it may be due to the fact that more fibula hypertrophy is needed to match the large diameter discrepancy in humerus as well as higher biomechanical stresses which occur there relative to the forearm [16]. 

Although a high complication rate may be anticipated, the free vascularized fibula may be used to reconstruct large skeletal defects in the pediatric upper extremity after oncologic resection. Complications may include nonunion and fracture, both of which occur more frequently in the humerus. We advocate for the use of compression plate fixation at osteosynthesis sites to prevent nonunion and careful protection of the extremity to prevent fracture, especially, when the humerus has been reconstructed. The vascularized fibular graft performs very well in reconstructing large skeletal defects in the pediatric upper extremity. Vascularized physeal transplant is a viable option when longitudinal growth is desired. 

## Figures and Tables

**Table 1:** Demographics.

Patient	Age	Sex	Diagnosis	Resected limb	Defect length (cm)	Chemotherapy	Primary fixation type	1° or 2° reconstruction	Mets	Flap type	Followup (mo)
1	17	Female	GCT	Distal radius	7	No	Plate and screws	Primary	No	OC	44
2	13	Male	Ewing sarcoma	Distal ulna	14	Yes	Plate and screws	Primary	No	OC	24
3	6	Male	Osteosarcoma	Humerus	8.3	Yes	Screws	Primary	No	Osseus	148
4	12	Male	Osteosarcoma telangiectasia	Distal radius	9	Yes	Plate and screws, wrist fusion	Unkn	No	Osseus	81
5	7	Male	Myxoid chondrosarcoma	Humerus	12	Unknown	External fixature	Secondary	No	OC	131
6	13	Female	GCT	Distal humerus	8.5	No	Plate and screws, K-wire, elbow fusion	Primary	No	Osseus	36
7	12	Male	Osteosarcoma	Humerus	20	Yes	K-wire	Primary	No	Osseus	24
8	4	Male	Osteosarcoma	Humerus	11	Yes	1 screw	Secondary	No	Osseus	23
9	11	Male	Osteosarcoma	Humerus/shoulder	15	Yes	Plate and screws	Primary	No	Osseus	16
10	6	Female	Osteosarcoma	Humerus	15	Yes	1 screw	Primary	Yes	Osseus	14.5
11	10	Male	Osteosarcoma	Proximal humerus	15	Yes	Plate and screws	Primary	Yes	OC	84

OC: Osteocutaneous, GCT: Giant cell tumor.

**Table 2:** Summary of complications.

Complication	Number of patients
Flap loss	0
Nonunion	3
Fracture of graft	3
Infection	0
Wound dehiscence	1
Hypertrophic scar	1
Fibular hardware failure	1
Peroneal n. palsy	4
	
Total patients with complications	**8**
Total patients requiring reoperation	**5**

**Table 3:** Measures of growth.

	Hypertrophy index (%)	Longitudinal growth (cm/year)
Diaphyseal transfers	55.7 (21–142)	(−)0.37 (−1.06–0.03)
Epiphyseal transfers	53.2 (35–89)	1.72 (0.13–4.68)
